# Dissecting the role of TRPV1 in detecting multiple trigeminal irritants in three behavioral assays for sensory irritation

**DOI:** 10.12688/f1000research.2-74.v1

**Published:** 2013-03-05

**Authors:** CJ Saunders, Winston Y Li, Tulsi D Patel, Jeffrey A Muday, Wayne L Silver

**Affiliations:** 1 Department of Biology, Wake Forest University, Winston-Salem, NC, 27109, USA; 2 Rocky Mountain Taste and Smell Center, Neuroscience Program, Department of Cell and Developmental Biology, University of Colorado, Anschutz Medical Campus, Aurora, CO, 80045, USA

## Abstract

Polymodal neurons of the trigeminal nerve innervate the nasal cavity, nasopharynx, oral cavity and cornea. Trigeminal nociceptive fibers express a diverse collection of receptors and are stimulated by a wide variety of chemicals. However, the mechanism of stimulation is known only for relatively few of these compounds. Capsaicin, for example, activates transient receptor potential vanilloid 1 (TRPV1) channels. In the present study, wildtype (C57Bl/6J) and TRPV1 knockout mice were tested in three behavioral assays for irritation to determine if TRPV1 is necessary to detect trigeminal irritants in addition to capsaicin. In one assay mice were presented with a chemical via a cotton swab and their response scored on a 5 level scale. In another assay, a modified two bottle preference test, which avoids the confound of mixing irritants with the animal’s drinking water, was used to assess aversion. In the final assay, an air dilution olfactometer was used to administer volatile compounds to mice restrained in a double-chambered plethysmograph where respiratory reflexes were monitored. TRPV1 knockouts showed deficiencies in the detection of benzaldehyde, cyclohexanone and eugenol in at least one assay. However, cyclohexanone was the only substance tested that appears to act solely through TRPV1.

## Introduction

Chemesthesis, the ability to detect chemical stimuli by the somatosensory system, enables the avoidance of potentially dangerous substances and is of considerable importance to an organism’s survival. Nociceptive fibers originating in both the dorsal root ganglia (DRG) and trigeminal ganglia (TG) respond to a variety of chemesthetic stimuli ^1^. In most mammals, nociceptive DRG fibers are protected from chemesthetic compounds by a keratinized epithelium ^2, 3^. Therefore, chemesthetic sensations are primarily mediated by trigeminal fibers that innervate the mucus membranes of the nasal cavity, nasopharynx, oral cavity and cornea. Additionally, chemesthetic agents can also stimulate vagal nerve fibers innervating the gut and respiratory tract. A wide variety of chemical irritants stimulate the polymodal nociceptors of the trigeminal nerve ^4^. While our knowledge of the receptors found on these nociceptive neurons is expanding, the mechanism of stimulation is known for only a few of these trigeminal chemical stimuli. For example, capsaicin, a compound found in chili peppers, activates transient receptor potential vanilloid 1 (TRPV1) channels ^5^.

TRPV1 is a member of the transient receptor potential (TRP) family of nonspecific cation channels ^6^, which were first identified in the *Drosophila* visual system ^7^. TRP channels are found in a multitude of mammalian tissues and are of particular importance in sensory systems ^8^. TRPV1 is activated by stimuli as diverse as plant metabolites, such as capsaicin ^5^, eugenol ^9^ and resiniferatoxin ^10^; low pH ^5^; temperatures above 43°C ^5^; endocannabinoids, such as anandamide ^11^; lipid-derived secondary messengers such as phosphoinositides ^12^; and metal ions, such as Ni ^2+^ ^13^. The channel is highly expressed by Calcitonin gene-related peptide- and substance P-positive nociceptive fibers originating in the DRG and TG ^14^. In addition to TRPV1 and TRPV family members 2–4, the nociceptive Aδ and C fibers of the trigeminal nerve also express TRP ankyrin 1 (TRPA1), TRP melastatin 8 (TRPM8), acid sensing ion channels (ASIC) and nicotinic acetylcholine receptors (nAChR) ^2^, which mediate chemical sensitivity to allyl isothiocyanate ^15^, menthol ^16^, acids ^17, 18^ and nicotine ^19^.

Fibers from the ophthalmic branch of the trigeminal nerve, cranial nerve V _1_, innervate the most rostral portion of the airway. Stimulation of these fibers results in protective airway reflexes, including coughing, vasodilation and decreased respiration rate ^1, 20, 21^. Chemesthetic compounds activate the trigeminal nerve either through direct stimulation of free nerve endings or by stimulation of solitary chemosensory cells. However, TRPV1 is found only on the free nerve ends and not on solitary chemosensory cells ^3, 21, 22^.

These facts support the concept that the trigeminal system is a warning system tuned to be responsive to diverse classes of potentially dangerous substances ^23^. As would be expected in such a warning system, the trigeminal system exhibits many aspects of redundancy, such as the presence of three different receptors that are sensitive to low pH: TRPV1, TRPA1 and ASIC ^18^ and responsiveness to very different chemical stimuli, indicated by the presence of multiple broadly tuned receptor proteins, such as TRPV1 and TRPA1. Much of the difficulty in determining the mechanism of stimulation of trigeminal irritants can be attributed to the redundancy and promiscuity of the trigeminal system.

To determine whether TRPV1 is necessary for the detection of trigeminal irritants other than capsaicin, we tested wildtype and TRPV1 knockout mice ( *Trpv1* ^–/–^) in three different behavioral assays for trigeminal irritation. In one assay, mice were presented with a chemical via a cotton swab and their responses scored on a 5-level scale. In another assay, a modified two-bottle preference test, which avoids mixing irritants with the animal’s drinking water to prevent post-ingestive effects, was used to assess aversion. In the final assay, a custom-built air-dilution olfactometer was used to administer volatile compounds to mice restrained in a double-chambered plethysmograph where respiratory reflexes were monitored, this representing a modified “Alarie Test” ^24, 25^. Our data suggest that, although many compounds may activate TRPV1, only a few, such as cyclohexanone, are exclusively detected by the channel, a finding that underlies the biological redundancy typified by the trigeminal system.

## Materials and methods

### Animals

Adult, female, wildtype (C57Bl/6J) and TRPV1 knockout mice (B6.129X1-Trpv1 ^tm1Jul^/J) were purchased from Jackson Laboratories in Bar Harbor, ME, USA, for the present study. Animals were given at least one week to acclimate after delivery to the Winston Hall animal facility. The mice were housed in conventional shoebox-type polycarbonate caging, changed once every 7 days. The bedding material was 1/8” Bed-o-cob, and they were provided with ad-lib water and rodent chow (Purinalab 5P00). Nesting material was provided in the form of Enrich-Nest. All animals were housed in groups of 4, provided with food and water *ad libitum* and were maintained on a 12 hour light cycle. 16 wildtype and 16 *Trpv1* ^-/-^ mice were used in this study; each mouse was tested in each experimental paradigm. At the end of the experiment, mice were tail biopsied and PCR was used to confirm the presence or absence of TRPV1. TRPV1 forward primer (5´-CGA GGA TGG GAA GAA TAA CTC ACT G-3´) and reverse primer (5´-GGA TGA TGA AGA CAG CCT TGA AGT C-3´) were purchased from Operon Biotechnologies (Huntsville, AL, USA). All experimental procedures were approved by Wake Forest University’s Animal Care and Use Committee (ACUC protocol # A07-104).

### Chemicals

Compounds tested included acetic acid, amyl acetate, benzaldehyde, capsaicin, cyclohexanone, eugenol (4-allyl-2-methoxyphenol), (-)-nicotine and toluene. All chemicals were purchased from Sigma Chemical Co. (St Louis, MO, USA) and had been certified ≥99% pure.

### Cotton swab test

The cotton swab test has been used previously to test the aversion of TRPA1 knockout mice to a number of chemicals ^25^. For liquid stimuli, cotton applicators (Puritan Medical Products, Guilford, ME, USA) were saturated with the pure compound before presentation to the animal. For powdered stimuli, the applicator was saturated with distilled water then placed in the compound so that it would adhere to the cotton.

To prevent the animals from becoming conditioned to associate the cotton swabs with aversive stimuli, several non-aversive substances were also presented to the mice. The favorable substances included chocolate powder (Nesquick, Glendale, CA, USA), water and wheat flour (King Arthur, Norwich, VT, USA); the noxious compounds tested were acetic acid, amyl acetate, benzaldehyde, capsaicin, cyclohexanone, eugenol, nicotine and toluene. Presentation was alternated between aversive and rewarding stimuli and an individual’s first three responses to the noxious substances were recorded.

Each animal was presented with each compound three times and its reaction to the stimulus was scored as follows:

-2: Rapid, reflexive withdrawal, removing the head from the stimulus source
-1: Rejection of stimulus marked by a slow head turn movement
- 0: No response or lack of interest in stimulus presentation
- +1: Investigation of stimulus source, marked by an increase in inspiratory sniffing
- +2: Attempted manipulation of stimulus source or attempted feeding behavior.

We have previously used this assay to assess the response of TRPA1 knockouts ^25^ and capsaicin-desensitized rats ^26^ to trigeminal irritants. For an individual mouse, a mean behavioral score was calculated for each substance and the scores from each animal were then averaged to ensure normally distributed data. To detect differences between the responses of wildtype and TRPV1-/- mice, a two-way ANOVA followed by Bonferroni post hoc tests was conducted with GraphPad Prism 5.03 for Windows (GraphPad Software, San Diego, CA, USA).

### Two-bottle preference test

Felt washers (Duro-Felt Products, Little Rock, AR, USA) were placed over the sipper tube of two water bottles and protected with a wire screen. The wire screen prevented the mouse from manipulating the washers or coming into direct contact with the compounds on the washers. One washer was soaked in undiluted irritant. The washer on the opposite tube was saturated with distilled water in place of irritant but otherwise prepared identically. No chemicals were mixed with the drinking water in either bottle. To approach and drink from a water bottle the mouse had to inhale the vapors created as the volatile compounds evaporated from the felt washer. Previous studies have used a traditional two-bottle preference paradigm where the capsaicin was mixed with the drinking water ^27^. While the traditional approach has the advantage of being able to test non-volatile compounds, such as the prototypical TRPV1 agonist capsaicin, it also allows the chemical to stimulate the taste system and possibly stimulate sensory fibers in the gut. Our modified two-bottle test avoids these confounding issues.

Individual mice were housed with two of the drinking tubes and water consumption was measured in each over 24 hrs. After an initial recording period of 24 hrs, the washers were renewed and the locations were switched to control for side bias. Water consumption was then measured over another 24 hrs. The values from the irritant-treated tube on the two days were summed and normalized to the total amount of water consumed (water consumed from irritant treated tube / (water consumed from irritant treated tube + water consumed from untreated tube). Tubes were of identical construction and all individual drinking tubes were tested over night before the experiment to ensure that they did not lose water through dripping. Data underwent an arcsine transformation to meet the assumptions of a two-way ANOVA. A two-way ANOVA followed by Bonferroni post hoc tests was conducted with GraphPad Prism 5.03 for Windows (GraphPad Software, San Diego, CA, USA) to detect differences between wildtype and TRPV1-/- mice.

### Respiratory assay

**Figure 1.**
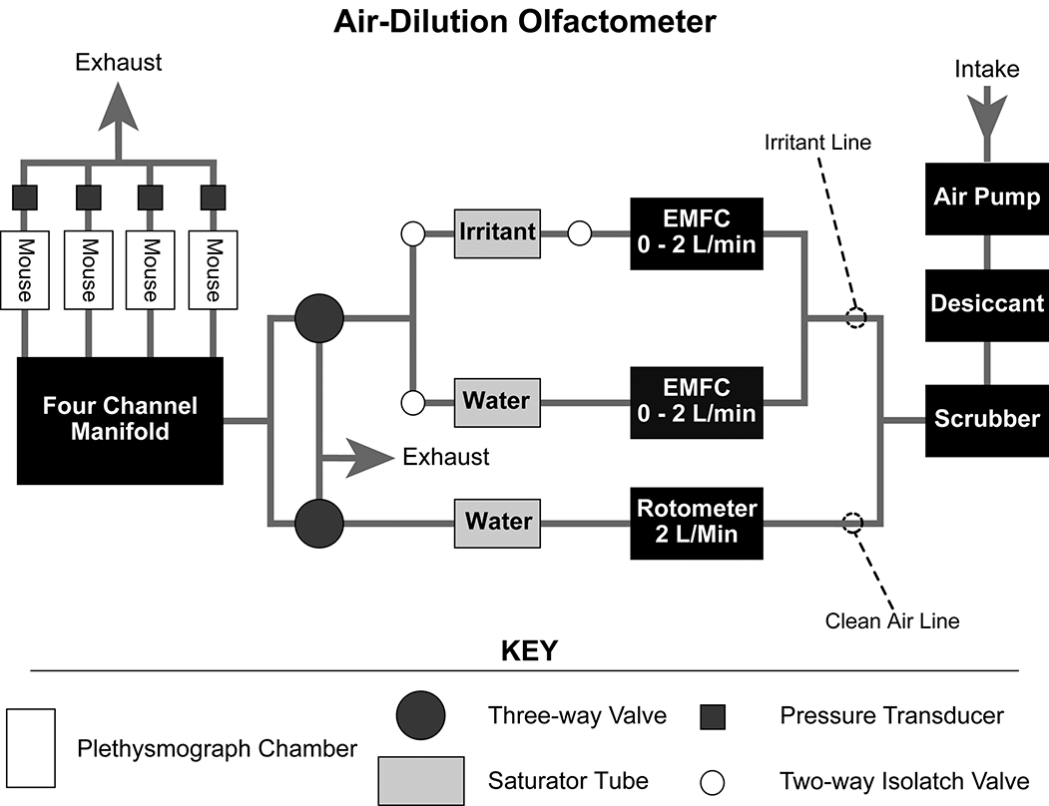
Diagram of the computer-controlled air-dilution olfactometer constructed to conduct the respiratory assay. EMFC, electronic mass flow controller.

A custom built, relatively inexpensive computer-controlled air-dilution olfactometer was used to administer volatile compounds to unanesthetized mice restrained in four double-chambered plethysmographs (Kent Scientific Corp, Torrington, CT, USA; Figure 1). The plethysmographs monitored the respiratory cycle via pressure transducers. Polytetrafluoroethylene tubing, 3/16” in diameter (Cole-Parmer, Vernon Hills, IL, USA), was used to connect all the individual components of the olfactometer and to construct the four-channel manifold.

The olfactometer was constructed so that air from a compressor was fed through desiccant and into a charcoal scrubber. This clean dry air flowed to two different lines, the clean airline and the irritant line. In the clean air line, air flowed to a glass rotometer (Cole-Parmer, Vernon Hills, IL, USA) set for a flow rate of 2 l/min. The air was humidified by passage through a saturator tube filled with deionized water. Humidification prevented dry air from desiccating the nasal mucosa of the mice. This line finally fed into a ganged three-way solenoid valve (Nacom Industries, Tustin, CA, USA) and provided clean air to mice at all times except for the stimulation period.

The irritant line was divided into two subdivisions, each of which was connected to an electronic mass flow controller (EMFC) (Sierra Instruments Inc, Monterey, CA, USA). One of these subdivisions fed into a saturator tube filled with distilled water, while the other fed into a saturator tube filled with undiluted liquid irritant. On both ends of the irritant saturator tube there was a two-way isolatch solenoid valve (General Valve Corporation, Pine Brook, NJ, USA) that controlled the forward flow and prevented backflow of the irritant. The saturator tube containing distilled water only required one valve to control forward flow because backward contamination was not a concern in this case. The two subdivisions of the irritant air line joined and led into a second ganged three-way solenoid valve. By using the two EMFCs to control the ratio of irritant saturated air to clean air, it was possible to control the concentration of irritant to which the animals were exposed. To maintain the air at a constant temperature, all saturator tubes were placed in a 21°C water bath. The irritant-laden air produced by the EMFCs flowed at a rate of 2 l/min.

Since the trigeminal nerve is polymodal and responds to mechanical as well as chemical stimuli ^1^, it is important to have a smooth transition between the clean air and the irritant-laden air. This was accomplished by smoothly switching the ganged three-way valves so that the clean air line flowed out via the exhaust and the irritant air line air flowed towards the mice. Air was sent through the EMFCs to produce the irritant 1 min before it was delivered to the mice in order to prevent excessive loss of the chemical in the saturator tube. Manipulation of the hardware was accomplished by writing a computer program in Visual Basic™ (program available below) which controlled a USB multi-Function data acquisition module from B & B Electronics (Model UD 128A8D; Ottawa, IL, USA) that was wired to the hardware.

Olfactometer control program Visual BasicTM computer control program for custom built air-dilution olfactometer.

The output of both the three-way solenoid valves was connected to a four-channel manifold that equally divided the air flow among the four plethysmograph chambers. The manifold was constructed from the same Teflon tubing used to connect the individual components of the olfactometer. By maintaining constant flow rates in the chambers, the pressure difference caused by inhalation by the test mouse provided sufficient pressure change to be used as an indicator of respiration. Each chamber was divided by a plastic ring, around which a latex dam was fitted, which formed a seal around each animal’s neck. The chambers were then connected to a pressure transducer (Kent Scientific, Torrington, CT, USA) which monitored the pressure change due to respiration. The outputs of the transducers were recorded using Acqknowledge 3.73 software (BIOPAC Systems, Goleta, CA, USA).

Respiration rate depression for female wildtype (C57Bl/6J) mice was calculated with AcqKnowledge and compared with that of female TRPV1 knockout mice for a variety of compounds in an attempt to determine whether TRPV1 was responsible for the detection of these irritants. Baseline respiration was recorded for 5 min. Respiration was then recorded for 5 min during irritant exposure. Wildtype and TRPV1-/- mice were sampled once per chemical at one concentration. Four mice were tested at the same time, each isolated in a separate plethysmograph chamber unable to interact with the other mice. The concentrations of amyl acetate tested were 2400 ppm, 2800 ppm, 3200 ppm and 4000 ppm. The concentrations of cyclohexanone tested were 1200 ppm, 1600 ppm, 2000 ppm and 3000 ppm. The concentrations of eugenol tested were 10 ppm, 15 ppm, 20 ppm and 25 ppm. The concentrations of nicotine tested were 10 ppm, 20 ppm, 30 ppm and 40 ppm. Concentrations were determined by using the Clausius-Clapeyron equation as previously described by Bryant and Silver ^1^. A two-way ANOVA followed by Bonferroni post hoc tests was conducted for each chemical with GraphPad Prism 5.03 for Windows (GraphPad Software, San Diego, CA, USA) to detect differences between wildtype and TRPV1-/- mice.

## Results

### Cotton swab test

**Figure 2.**
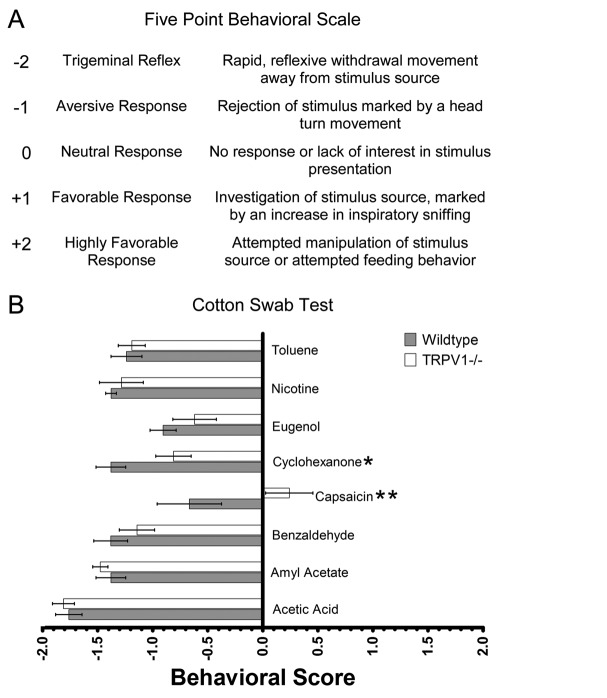
TRPV1 knockout mice are significantly less averse to capsaicin and cyclohexanone than wildtype mice in an acute presentation. ( **A**) A five-point scale ranging from -2 for a trigeminal reflex to +2 for a highly favorable response was used to assign behavioral scores for each substance. ( **B**) Mean ± SEM behavioral score for wildtype mice ( *n*=7), filled bars, and TRPV1-/- knockout mice ( *n*=7), unfilled bars. Two-way ANOVA revealed a significant difference among irritants ( *P*<0.001), genotype ( *P*<0.01) and a significant interaction between irritant and genotype ( *P*<0.05). Knockout mice had a significantly more favorable response to capsaicin ( *P*<0.01) and cyclohexanone ( *P*<0.05) than wildtype mice. *P<0.05, **P<0.01, ***P<0.001.

Cotton swabs saturated with an irritant were presented to wildtype (n=7) and TRPV1 (n=7) knockout mice to determine whether TRPV1 is necessary for detection of an acute presentation of an irritating compound. The response to these compounds was scored on a five-point scale. The scale ranged from -2 for a reflexive trigeminal response, to 0 for no response to the cotton swab, to +2 for feeding behavior (summarized in Figure 2A). Two-factor ANOVA revealed a significant difference between irritants ( *P*<0.001), genotype ( *P*<0.01) and a significant interaction between irritant and genotype ( *P*<0.05).

Wildtype mice showed aversive responses to all noxious substances ( Figure 2B). Acetic acid produced the most pronounced aversive response of all the substances tested, typically a distinct trigeminal reflex followed by the animal retreating from the cotton swab. Amyl acetate, benzaldehyde, cyclohexanone, nicotine and toluene all produced aversive behaviors in the wildtype mice, although the responses were not as extreme as the response to acetic acid. Capsaicin and eugenol were both slightly aversive but did not produce responses as extreme as those induced by the other noxious compounds.

The response of TRPV1 knockout mice to most substances tested was similar to the response of wild type mice ( Figure 2B). The only substances to which TRPV1 knockout mice showed significantly less aversion to than the wildtype mice were capsaicin (p<0.01) and cyclohexanone (p<0.05). The response of the TRPV1 knockouts to capsaicin was in the non-aversive range. However, the response of the TRPV1 knockout mice to cyclohexanone was still in the aversive range. There appeared to be some divergence in the response of TRPV1 knockouts to eugenol but statistical analysis revealed these disparities were not significant.

### Two bottle preference test

In this assay, mice were able to control their exposure to vapors of the chemical irritant being tested, unlike the cotton swab and respiratory assays, where mice were exposed to a chemical irritant and their behavior monitored. In previous two-bottle preference experiments with trigeminal irritants, the chemicals were mixed with the drinking water ^27^. A disadvantage of this approach is that it might result in stimulation of gustatory or gut chemosensors in addition to the trigeminal system ^28^. We avoided mixing chemicals with the drinking water by treating felt washers with the chemical irritants and placing these washers over the sipper tube of the water bottles. However, a disadvantage of our approach is that the non-volatility of capsaicin prevented it from being tested.

If TRPV1 alone is required to detect a noxious chemical then TRPV1 knockout mice should not avoid water bottles surrounded by vapors of that chemical. A control experiment conducted without irritant applied to the washers on either drinking tube (both washers treated with distilled water) indicated no significant side bias and no differences between the amount of water consumed by wildtype and TRPV1 knockout mice (data not shown).

**Figure 3.**
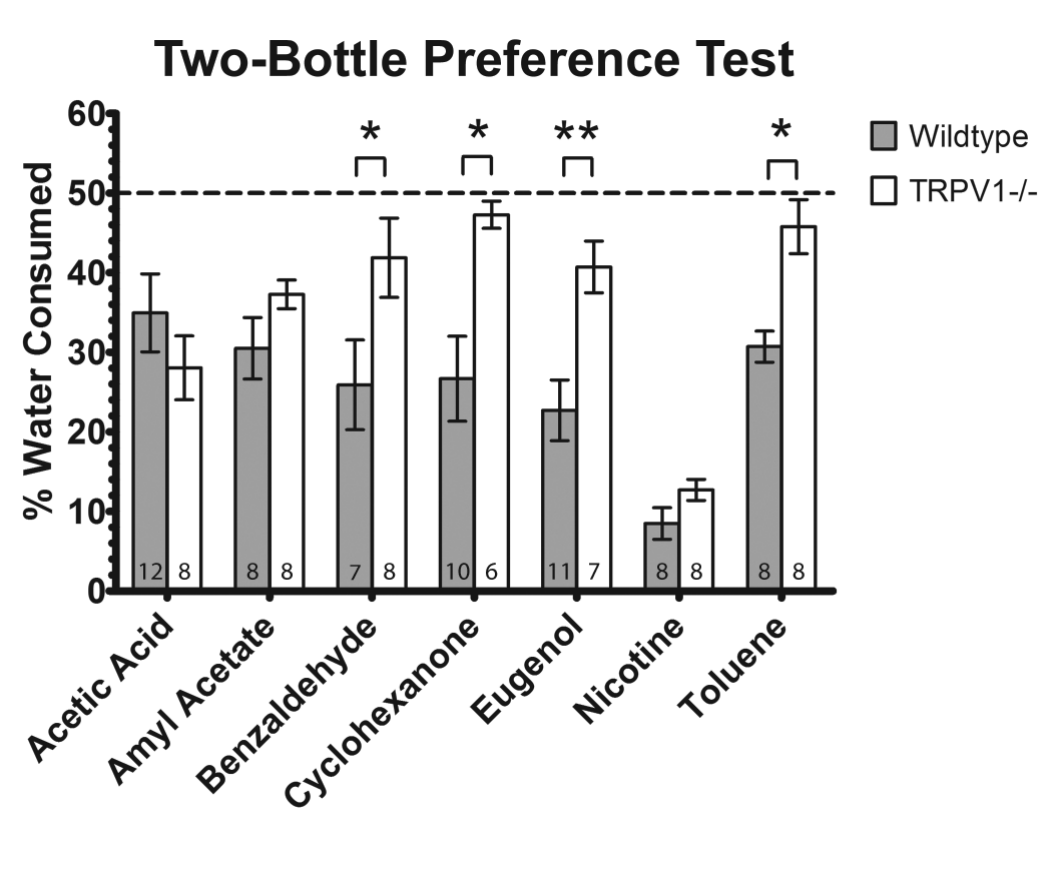
TRPV1 knockout mice show significant deficiencies in the ability to detect benzaldehyde, cyclohexanone, eugenol and toluene. Results are from the two-bottle preference test. Mean ± SEM percentage of water consumed from the irritant-treated tube by wildtype and TRPV1-/- mice. Two-way ANOVA revealed a significant difference among irritants ( *P*<0.001), genotype ( *P*<0.001) and interaction between the two factors ( *P*<0.01). Knockout mice drank significantly more than wildtype mice from bottles treated with benzaldehyde ( *P*<0.05), cyclohexanone ( *P*<0.05), eugenol ( *P*<0.01) and toluene ( *P*<0.05). The number of mice in each experimental group is indicated at the foot of each bar. *P<0.05, **P<0.01, ***P<0.001.

To compare water consumption between wildtype and TRPV1 knockout mice, the mean and SEM water consumption values from the irritant-treated tube were normalized to the total amount of water consumed from both bottles. TRPV1 knockout mice drank significantly more water from bottles treated with benzaldehyde (p<0.05), cyclohexanone (p<0.05), eugenol (p<0.01) and toluene (p<0.05) than wildtype mice ( Figure 3). There was no significant difference in the amount of water consumed from bottles treated with acetic acid, amyl acetate and nicotine between wildtype and TRPV1 knockout mice. Two-way ANOVA also revealed a significant difference between irritants ( *P*<0.001), genotype ( *P*<0.001) and interaction between the two factors (genotype and irritant, p<0.01).

### Respiratory assay

**Figure 4.**
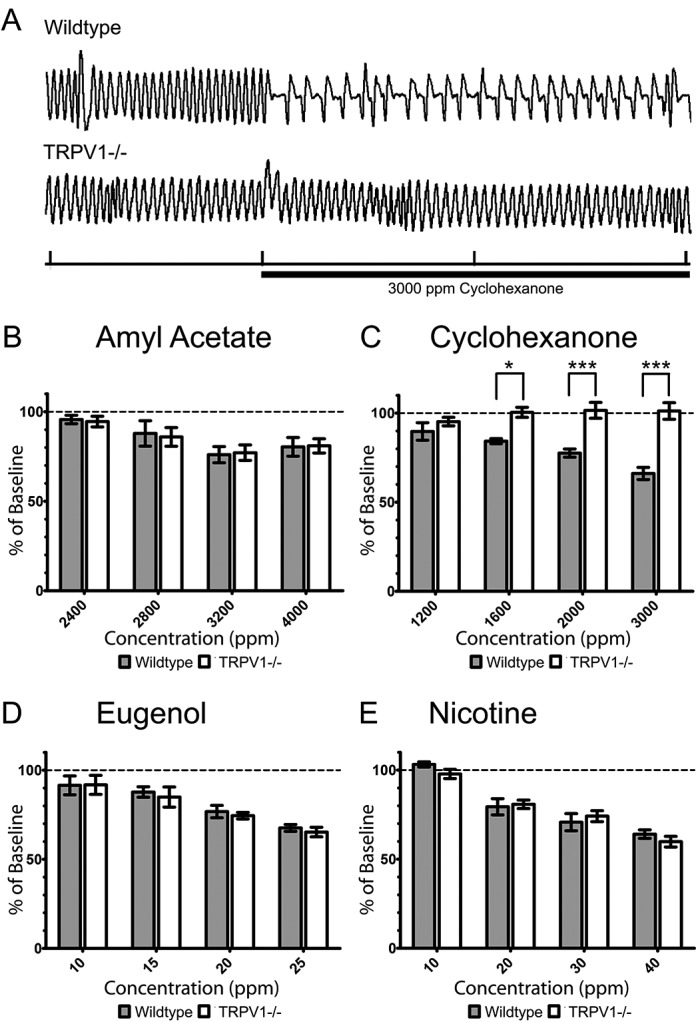
Respiration rate depression in response to trigeminal irritants. ( **A**) Sample traces from a wildtype and TRPV1 knockout mouse in a double-chambered plethysmograph showing the typical respiratory response to 3000 ppm cyclohexanone (marked by horizontal bar). Tick marks on the *x*-axis indicate 6 sec. ( **B**– **E**) Percent respiratory depression of four animals expressed as mean ± SEM ( *n*=4). ( **B**) The effect of amyl acetate exposure was not significantly different between wildtype and knockout mice. A two-way ANOVA detected a significant difference between concentrations ( *P*<0.01) but no significance between genotype or interaction between the two. ( **C**) The effect of cyclohexanone exposure was significantly different between wildtype and knockout mice at concentrations of 1000 ppm ( *P*<0.05), 2000 ppm ( *P*<0.001) and 3000 ppm ( *P*<0.001). A two-way ANOVA detected a significant difference between genotype ( *P*<0.001) and a significant interaction ( *P*<0.01) but no significant difference between concentration. ( **D**) The effect of eugenol exposure was not significantly different between wildtype and knockout mice. A two-way ANOVA detected a significant difference between concentrations ( *P*<0.001) but no significance between genotype or interaction between the two. Finally, ( **E**) the effect of nicotine exposure was not significantly different between wildtype and knockout mice. A two-way ANOVA detected a significant difference between concentrations ( *P*<0.001) but no significance between genotype or interaction between the two. *P<0.05, **P<0.01, ***P<0.001.

Since many trigeminal irritants are also detectable by the olfactory system, the aversion to chemicals observed in the cotton swab and two-bottle preference tests could possibly be due to detection by that system. To overcome this uncertainty, an assay of sensory irritation was needed that was specific to trigeminal irritation. Trigeminal stimulation causes stereotyped changes to the normal exhalation pattern. Specifically, trigeminal stimulation results in a reflexive increase in airway resistance and is characterized by an increase in the period of initial expiration ( Figure 4A). This “braking” during the first stage of expiration results in a longer period between breaths and thus effectively decreases respiratory frequency and has been used extensively as an assay of trigeminal irritation ^21, 22, 27, 29– 32^. A typical trace of baseline respiration and the response to 3000 ppm cyclohexanone from a wildtype and TRPV1 knockout mouse are shown in Figure 4A. To compare respiration rate depression between wildtype and TRPV1 knockout mice, the respiration rate during the 5 min irritant exposure was normalized to the 5 min baseline recording.

Amyl acetate ( Figure 4B), eugenol ( Figure 4D) and nicotine ( Figure 4E) caused respiratory rate depression in wildtype and TRPV1-/- mice in a dosage-dependent manner. There were no significant differences in the respiratory rate depression seen in wildtype and TRPV1-/- mice at any concentration of amyl acetate, eugenol or nicotine. There was a significant difference in the respiratory depression seen between different concentrations of amyl acetate ( *P*<0.01), eugenol ( *P*<0.001) and nicotine ( *P*<0.001) but no significant differences were detected between genotype or from interaction between genotype and concentration for these compounds.

A significant interaction was detected between genotype and cyclohexanone concentration ( *P*<0.01) by two-way ANOVA. There was also a significant difference between genotype ( *P*<0.001) but not concentration. There was no significant difference between the respiration rate of wildtype and TRPV1 knockout mice at 1200 ppm cyclohexanone, the lowest concentration tested. At higher concentrations, the respiration rate of wildtype mice was significantly lower than TRPV1 ^-/-^ mice at 1600 ppm ( *P*<0.05), 2000 ppm ( *P*<0.001) and 3000 ppm ( *P*<0.001) cyclohexanone ( Figure 4C). Cyclohexanone depressed the respiration rate of wildtype mice in a dosage-dependent manner. However, cyclohexanone did not alter the respiration rate of TRPV1 knockout mice from its baseline at any concentration.

Ability of TRPV1 knockout mice to detect trigeminal irritants Raw data tables showing the response of wild type and Trpv1 knockout mice in the cotton swab test, two-bottle preference test and respiratory assay.

## Discussion

Polymodal nociceptive trigeminal fibers are sensitive to a wide range of chemical stimuli ^4^. This sensitivity is due to the expression of a diverse population of receptor proteins, many of which are expressed by the same fiber ^2^. For example, TRPV1 and TRPA1 are coexpressed by the same trigeminal fibers ^33^. This arrangement of receptors is largely responsible for the broad responsiveness of the trigeminal nerve and also provides redundant detection mechanisms for many chemicals. Understanding these features of the trigeminal system is of considerable importance in understanding its role as a broadly tuned warning system of potentially dangerous compounds ^23^. Additionally, the presence of multiple broadly responsive receptors with overlapping sensitivity has made it difficult to determine the exact mechanisms by which a chemical stimulates trigeminal fibers and represents a profound experimental challenge. In the present study, we have taken advantage of the availability of TRPV1 knockout mice to elucidate the role of TRPV1 in detecting several known trigeminal irritants.

### Capsaicin

Capsaicin, the active component of chili peppers, was used to clone the TRPV1 channel ^5^ and has been used as a topical TRPV1 agonist in a multitude of studies ^34– 36^. While capsaicin is the prototypic exogenous TRPV1 agonist, the non-volatility of capsaicin restricts its use in experimental paradigms where the stimulus is administered as a vapor. Our previous experiments established capsaicin as a potent trigeminal irritant, but administration to the upper respiratory tract required surgical manipulation ^4^. The current study examines the involvement of TRPV1 in the detection of trigeminal irritants in unanesthetized and minimally restrained animals. This approach has the benefit of more accurately simulating how terrestrial vertebrates, including humans, naturally encounter upper respiratory tract irritants but also highlights the complexity of using capsaicin in studies of upper respiratory tract irritation.

Capsaicin was tested in the cotton swab assay where TRPV1 knockout animals found it less aversive than wild type mice. This result is entirely consistent with the multitude of experiments indicating that TRPV1 is responsible for detecting capsaicin ^34– 36^. Wildtype mice found capsaicin to be the least aversive of all the irritants tested in this assay. This result supports the concept that despite the potency of capsaicin as a painful stimulus when applied topically, the poor volatility of the chemical prevents it from reaching the pain fibers of the upper respiratory tract under normal conditions.

### Nicotine

Nicotine occurs naturally in a number of plant species where it is thought to act as a repellent to potential predators. Humans have cultivated many of these plants to allow for the recreational use of nicotine ^37^. Previous work has established nicotine as a potent trigeminal irritant that is primarily detected via nicotinic acetylcholine receptors (nAChRs) ^19^ or TRPA1 ^38^. Since nicotine is known to have a mechanism of stimulation independent of TRPV1 ^19, 38^, it was included in this study to ensure that TRPV1 knockout mice respond normally to trigeminal irritants. In every case, the response of TRPV1 knockout mice to nicotine did not differ from wildtype mice, indicating that these mice were capable of detecting and responded to trigeminal irritants normally.

### Acetic acid

Acetic acetate is produced by a variety of bacteria during fermentation. Dilute acetic acid is commonly used as a flavor enhancer while concentrated acetic acid is used in a variety of industrial processes ^39^. Due to its low pH, acetic acid is a potent chemesthetic stimulus that is capable of activating both ASIC, TRPA1 ^18^ and TRPV1 ^40^ proteins on the trigeminal nerve ^2, 4^. TRPV1 knockout mice showed no deficiencies in their ability to detect concentrated acetic acid in either the cotton swab or the two-bottle preference tests. Previous experiments established that TRPV1 knockout mice show normal expiratory pause reflex in response to acetic acid ^20^. Presumably, trigeminal ASIC and TRPA1 receptors respond to acetic acid even in the absence of TRPV1, allowing the TRPV1 knockout mice to detect and avoid acids through this redundancy. Additionally, the olfactory system could be mediating or contributing to the avoidance of acetic acid in the cotton swab or the two-bottle preference tests.

### Amyl acetate

Amyl acetate occurs naturally in fruits such as bananas and pears. It is also produced synthetically for use as an artificial flavoring because of its distinctly fruity odor ^39^. In addition to stimulating the olfactory system, at high concentrations amyl acetate also stimulates the trigeminal system ^4, 25^. Previous work has demonstrated that while amyl acetate activates TRPA1 it is not the only mechanism by which amyl acetate is detected ^25^.

In the present study, TRPV1 knockout mice showed no deficiencies in their ability to detect and avoid amyl acetate in the cotton swab or the two-bottle preference tests. Additionally, amyl acetate induced the expiratory pause reflex to the same degree in TRPV1 knockout mice and wildtype mice. These findings are consistent with previous work that demonstrated that TRPV1 does not respond to amyl acetate *in vitro* ^4^. These results establish that TRPV1 is not one of the redundant mechanisms by which amyl acetate is detected.

### Benzaldehyde and toluene

Benzaldehyde occurs naturally in almonds and is produced synthetically to be used as a reactant and solvent in industrial chemistry ^39^. The sap of the tolu balsam tree contains toluene and has been used as an ingredient in cough syrups ^41^. Both benzaldehyde and toluene stimulate the trigeminal nerve ^4, 24^, fail to activate TRPV1 *in vitro* ^4^, activate TRPA1 *in vivo* and fail to induce the expiratory pause reflex in TRPA1 knockouts ^25^. However, TRPA1 knockouts find benzaldehyde less aversive in the cotton swab paradigm but respond to toluene normally ^25^. In the current study, TRPV1 knockouts respond normally to an acute presentation of benzaldehyde and toluene in the cotton swab test but drink significantly more from drinking tubes treated with either chemical than wildtype mice. These contradictory results may indicate that both TRPV1 and TRPA1 are required for the normal detection of these chemicals. If both these chemicals are irritating the respiratory epithelium via trigeminal TRPA1 channels then the subsequent tissue inflammation will result in increased levels of prostaglandins and bradykinin ^6^. Prostaglandins and bradykinin are known to sensitize TRPV1 channels ^42^ and it is possible that, when sensitized by these molecules, TRPV1 channels could be activated by benzaldehyde or toluene. Additionally, most TRPA1-expressing fibers also express TRPV1 ^33^. Stimulation of TRPA1 by benzaldehyde or toluene could lead to increased production of lipid-derived secondary messengers which can stimulate TRPV1 in the same fiber ^12^. Alternatively, interactions between the olfactory and trigeminal systems that involve both TRPV1 and TRPA1 may be responsible for the detection of these compounds ^25, 43– 45^. Future studies should consider the use of TRPV1/TRPA1 double knockout mice to explore this possibility that both channels are involved in the detection of benzaldehyde and toluene.

### Eugenol

Eugenol is a major component of clove oil. Eugenol has a number of uses in fragrance and flavor industries and is commonly used as a dental analgesic ^46^. Previous work has established that eugenol is capable of activating TRPV1 ^9^, TRPA1 and TRPM8 ^47^ *in vitro*. Eugenol induced a normal expiratory pause reflex in TRPV1 knockout mice. To our knowledge, this study is the first to demonstrate a eugenol-induced expiratory pause reflex. TRPV1 knockout mice showed no deficiencies in their ability to detect an acute presentation of eugenol in the cotton swab assay. These results are consistent with the presence of TRPA1 and TRPM8 being sufficient to mediate protective airway reflexes that are mediated solely though the trigeminal stimulation. However, TRPV1 knockout mice drank significantly more from drinking tubes treated with eugenol than wildtype mice. It is possible that eugenol stimulation activates TRPA1 and results in the sensitization of TRPV1 as described above.

### Cyclohexanone

Cyclohexanone is synthesized in large quantities for its application in the production of polymers ^48^ and has no known natural source ^49^. Exposure to residual cyclohexanone present in medical devices composed of polymers is thought to cause dangerous physiological effects ^49^. Cyclohexanone is a potent trigeminal irritant ^19^ and activates TRPV1 channels, but not TRPA1, *in vitro* ^4, 50^. TRPV1 knockout mice show a significant reduction in sensitivity to cyclohexanone in all three assays used in this study. Strikingly, TRPV1 knockout mice fail to alter their respiration rate when exposed to concentrations of cyclohexanone sufficient to depress respiration in wildtype mice by over 30% ( Figure 4C). While TRPV1 knockout mice find cyclohexanone significantly less aversive than wildtype mice in the cotton swab and two-bottle drinking assays, they still avoid cyclohexanone. This result is logically consistent with the concept that some chemicals stimulate both the olfactory and trigeminal systems. In the case of the cotton swab and two-bottle drinking assays, cyclohexanone can still be detected by olfactory receptors, allowing the animal to perceive and avoid the chemical. However, only the trigeminal system is involved in inducing the expiratory pause reflex ^21, 30^. TRPV1 knockout mice show no expiratory pause reflex when exposed to cyclohexanone, and therefore TRPV1 is necessary for cyclohexanone-induced sensory irritation.

## Conclusions

Acetic acid, amyl acetate, benzaldehyde, capsaicin, cyclohexanone, eugenol, nicotine and toluene are all potent trigeminal irritants. All of these compounds, except cyclohexanone, have botanical or bacterial sources that could easily be encountered by mammals. Cyclohexanone is commonly used in the synthesis of polymers, and exposure to residual cyclohexanone can have negative effects on human health. Of the eight compounds tested, five (acetic acid, amyl acetate, benzaldehyde, eugenol, and toluene) are thought to have redundant mechanisms of action. TRPV1 knockout mice showed deficiencies in their ability to detect each of these compounds in only one of the three assays of trigeminal irritation. Cyclohexanone was the only compound tested that was significantly less aversive to TRPV1 knockouts in all three assays. Furthermore, TRPV1 is required for the detection of cyclohexanone by the trigeminal system because TRPV1 knockout mice lacked respiratory reflexes in response to this compound. It is notable that redundant mechanisms used by the trigeminal system to detect the naturally occurring chemicals tested are also sufficient to detect at least one potentially dangerous compound that has no natural source, cyclohexanone.
