# Supplementary material for: Dissecting the role of TRPV1 in detecting multiple trigeminal irritants in three behavioral assays for sensory irritation
Source: F1000Res. 2013 Mar 5;2:74. [Version 1] doi: 10.12688/f1000research.2-74.v1 (PMC3814916; doi:10.12688/f1000research.2-74.v1)
Supplement: Peer review history

# Peer review: Referee response for version 1

**Published:** 2013-03-12

**Authors:** Gunnar D Nielsen

**Affiliations:** 1 National Research Centre for the Working Environment, Copenhagen, Denmark

The hypothesis is that trigeminal induced (sensory) irritation of chemicals is due to multiple receptors, which is summarized in the Introduction section. Thus an irritant response may be due to activation of several receptors providing redundant detection of an irritant effect of a chemical. 

This study investigates the role of the TRPV1 (“capsaicin receptor”) in activation of the trigeminal nerve by seven substances (toluene, nicotine eugenol, cyclohexanone, capsaicin, benzaldehyde, amyl acetate and acetic acid).

Mice with and without the TRPV1 receptor (wild type and knockout mice) were used. Each substance was studied in both types of mice by three methods: 

1) Withdrawal from a cotton swab soaked in the compound;

2) A two bottle drinking water preferred choice test, where the atmosphere around one of the bottles was clean air and the other was with the air containing the test compound, and; 

3) A reflex induced decreased in respiratory rate due to activation of the trigeminal nerve, a relatively specific method.

It was convincingly demonstrated that cyclohexanone was less irritating in all three test methods in the knockout mice which indicates that the irritation response at least partly was mediated by the PRPV1 receptor. In the specific respiratory rate test, the TRPV1 receptor was essential for its detection. 

The manuscript is consistent and well written. The last author (Wayne L Silver) is a pioneer within sensory irritation research.

I have read this submission. I believe that I have an appropriate level of expertise to confirm that it is of an acceptable scientific standard.

# Peer review: Referee response for version 1

**Published:** 2013-03-08

**Authors:** Bruce Bryant

**Affiliations:** 1 Monell Chemical Senses Center, Philadelphia, PA, USA

This is a nicely designed study, well executed. Since there were 3 assays used I was expecting more comparison of the 3 assays. Also, in the discussion is capsaicin a typical or topical TRPV1 agonist?

I have read this submission. I believe that I have an appropriate level of expertise to confirm that it is of an acceptable scientific standard.
